# Simplified Lead Detection: Graphene-Based Potentiometric Sensors for Pb(II) Monitoring

**DOI:** 10.3390/s26175395

**Published:** 2026-08-26

**Authors:** Martyna Drużyńska, Nikola Lenar, Beata Paczosa-Bator

**Affiliations:** Faculty of Materials Science and Ceramics, AGH University of Krakow, Mickiewicza 30, PL-30059 Krakow, Poland; druzynska@agh.edu.pl

**Keywords:** lead(II) ions, graphene, ion-selective electrode, single-piece electrode, lead detection, potentiometry

## Abstract

**Highlights:**

**What are the main findings?**

- A graphene-modified single-piece all-solid-state Pb(II)-selective electrode was successfully developed without the need for a separate solid-contact layer.
- Optimization of membrane thickness identified the optimal sensor architecture, while graphene incorporation improved charge-transfer properties and suppressed water-layer formation.

**What are the implications of the main findings?**

- Direct incorporation of graphene into the ion-selective membrane provides a simple and effective strategy for improving the performance of all-solid-state potentiometric sensors.
- The proposed sensor architecture offers an easy-to-fabricate platform for environmental monitoring of lead(II) ion.

**Abstract:**

Lead contamination remains a significant environmental and public health concern, creating a demand for analytical platforms that combine sensitivity, simplicity, and long-term stability. In this work, a graphene-containing molecular membrane matrix was developed for the fabrication of single-piece all-solid-state potentiometric sensors for Pb(II) detection. The sensing membrane consisted of poly(vinyl chloride), plasticizers, a Pb(II)-selective ionophore, lipophilic ionic sites, and dispersed graphene nanostructures, forming an integrated molecular sensing interface. Within the membrane phase, selective complexation of Pb(II) ions by the ionophore was coupled with graphene-assisted ion-to-electron transduction, enabling efficient signal generation without the need for a separate solid-contact layer. The influence of graphene incorporation and membrane thickness on sensor performance was systematically investigated. Among the tested configurations, a membrane prepared from 40 µL of sensing cocktail provided the best overall performance, combining high electrical capacitance, favorable surface properties, and superior potential stability. SEM imaging revealed a homogeneous membrane morphology without large graphene agglomerates, indicating effective dispersion of graphene within the polymer matrix. The optimized sensor exhibited a near-Nernstian slope of 30.3 mV dec^−1^, a linear response range from 1.0 × 10^−7^ to 1.0 × 10^−2^ M, a detection limit of 6.3 × 10^−8^ M, and a potential drift of only 0.35 mV h^−1^. These results demonstrate that direct incorporation of graphene into an ion-selective membrane is an effective strategy for constructing robust and scalable single-piece potentiometric sensors for Pb(II) monitoring and highlight the potential of developed membrane materials for electrochemical sensing applications.

## 1. Introduction

Lead (Pb) is classified as a heavy metal that poses a significant threat to the environment and human health. Lead exhibits high toxicity even at low concentrations [1,2,3]. Apart from natural sources of lead, such as volcanic eruptions and geochemical weathering, the prevalence of this element in the environment is largely associated with human activity [4,5]. Most of the contamination comes from industrial processes such as urbanization [6], the glass industry [7], mining [8], and wastewater discharge [9]. As a result, exposure to this heavy metal can lead to neurotoxicity, developmental delays in children, and anemia. Therefore, reliable monitoring of lead contamination is crucial [10,11,12]. Conventional analytical methods such as atomic absorption spectrometry (AAS) [13], inductively coupled plasma mass spectrometry (ICP-MS) [14], and anodic stripping voltammetry (ASV) [15] are used in environmental screening due to their high sensitivity and accuracy. Potentiometry has at an advantage over these methods. Potentiometric techniques allow for direct, on-site measurement of ionic activity with minimal sample processing.

Polymeric ion-selective membranes consist of components that perform complementary roles in the sensing process. Ionophores provide selective recognition of target ions, lipophilic ionic sites facilitate ion exchange, plasticizers control membrane mobility, and conductive additives may promote ion-to-electron transduction. The interactions among these constituents determine the analytical performance, stability, and selectivity of all-solid-state potentiometric sensors. Consequently, such electrodes offer a simple, cost-effective, and field-deployable approach to ion detection, making them particularly attractive for environmental analysis, where rapid on-site monitoring of trace contaminants is often required. The development of membrane matrices that integrate recognition and transduction functionalities within a single phase therefore represents an important direction in electrochemical sensing [16,17].

Ion-selective electrodes (ISEs) enable the detection of various analytes over a wide range of concentrations and have therefore become robust tools for ion analysis, including trace-level measurements. Among the available ISE architectures, all-solid-state electrodes are particularly promising due to their simple fabrication, easy miniaturization, and favorable analytical properties [16,17]. Single-piece (SP) electrodes presented by Bobacka et al. [18] are particularly noteworthy. They are characterized by simplified construction obtained by direct incorporation of active material into ion-selective membrane. The fabrication of such sensors is much simpler compared to solid-contact (SC) electrodes, with the additional step of manufacturing the intermediate solid-contact layer [19]. Therefore, the introduction of new materials creates opportunities for the development of single-piece electrodes. So far, materials such as conducting polymers [20,21], molecularly imprinted polymers (MIPs) [22], ionic liquids (IL) [23], monolayer-protected clusters (MPCs) [24] and carbon nanomaterials [25,26,27] were used as ion-to-electron transducers to fabricate single-piece electrodes.

Previous Pb(II)-selective potentiometric sensors have demonstrated near-Nernstian sensitivities and low detection limits. For example, a solid-contact electrode modified with a carbon nanofiber–ionic liquid nanocomposite exhibited a detection limit of 6 × 10^−9^ M [28], while an all-solid-state Pb(II)-selective electrode using bimodal-pore C_60_ as a solid contact showed a detection limit of 6 × 10^−9^ M [29]. These results demonstrate the feasibility of potentiometric Pb(II) detection at low concentration levels using different electrode architectures.

In this work, we developed a Pb^2+^-selective polymeric membrane with dispersed graphene (GR) to obtain single-piece electrodes without any additional layers. Plasticized poly(vinyl chloride) (PVC) was used as the membrane matrix due to its suitable physical properties [30]. The membrane preparation was optimized using different membrane thicknesses to investigate their effect on sensor performance. The membranes were applied onto glassy carbon (GC) disk electrodes, and the influence of graphene incorporation on the potentiometric performance of the resulting electrodes was investigated.

To the best of our knowledge, Pb(II)-selective single-piece electrodes, in which graphene is incorporated directly into the ion-selective membrane without a separate solid-contact layer, have not been previously reported. The proposed approach combines charge transduction and structural stabilization within a single membrane phase, thereby simplifying sensor fabrication while maintaining competitive analytical performance.

## 2. Materials and Methods

### 2.1. Chemicals

The ion-selective membrane (ISM) components included poly(vinyl chloride) (PVC) of high molecular weight, lipophilic salt potassium tetrakis (p-chlorophenyl) borate (KTpClPB), plasticizers 2-nitrophenyl octyl ether (*o*-NPOE) and bis(1-butylpentyl) adipate (BBPA), and lead ionophore IV. All components were dissolved in tetrahydrofuran (THF). All membrane components and the solvent were purchased from Sigma Aldrich, St. Louis, MO, USA. Commercially available single-layer graphene (GR), used as a membrane additive, was obtained from the Nanostructured & Amorphous Materials, Inc. (Houston, TX, USA).

As a standard lead ion solution, lead(II) nitrate aqueous solutions were used. Lead(II) nitrate (Pb(NO_3_)_2_) was purchased from Chempur (Piekary Śląskie, Poland). The standard Pb^2+^ ion solutions were used for all the conducted measurements, including potentiometry, electrochemical impedance spectroscopy, and chronopotentiometry. For the additional examination and validation of the designed sensors, potassium nitrate (KNO_3_), purchased from POCH (Gliwice, Poland), was used. For the preparation of aqueous solutions, distilled and deionized water was used. All chemicals were used as received, without any further purification.

### 2.2. Sensor Preparation

In this work, single-piece Pb(II)-selective electrodes were fabricated by depositing a sensing cocktail onto GC disk electrodes. The membrane consisted of lipophilic ionic sites, plasticizing agents, and graphene nanostructures dispersed within a polymeric environment, forming an integrated sensing phase responsible for selective ion recognition and ion-to-electron transduction.

**Figure 1:**
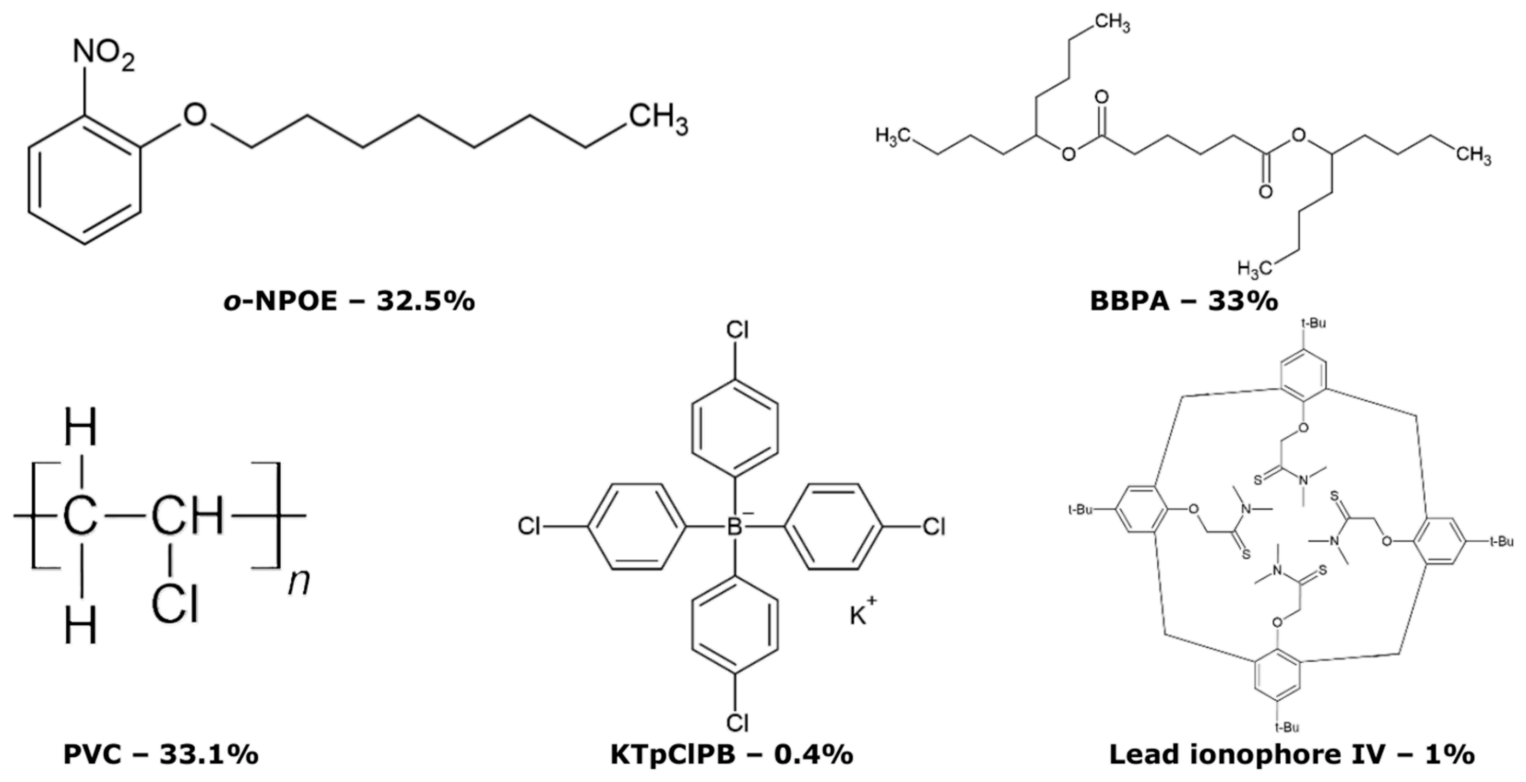
Individual components used to prepare the polymeric ion-selective membrane, presented in their respective weight percentages.

The polymeric membrane was prepared using (*w*/*w*%) polyvinyl chloride (33.1%), two types of plasticizers—bis(1-butylpentyl) adipate (BBPA) and 2-nitrophenyl octyl ether (o-NPOE) (respectively 33% and 32.5%), lead-selective ionophore IV (1%), and lipophilic salt (0.4%). All individual membrane components are presented in Figure 1. The dissolved membrane components were divided into two separate vials. In one formulation, graphene flakes (3.8 *w*/*w*%) were incorporated directly into the membrane matrix, whereas the second formulation, prepared without graphene, served as the control system.

**Figure 2:**
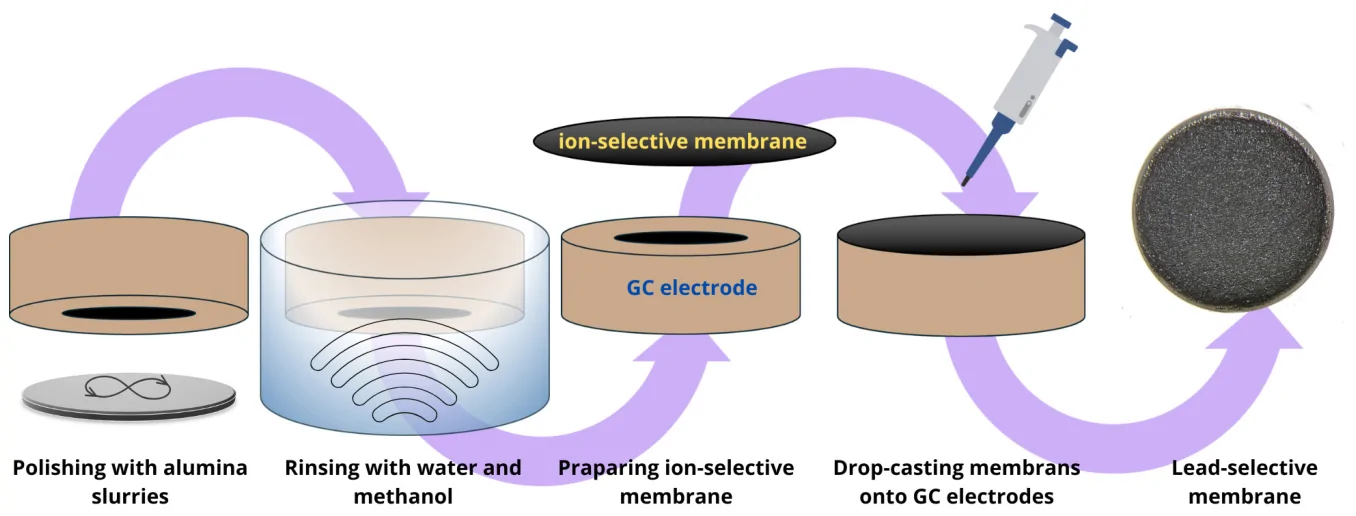
Schematic representation of simplified preparation procedure for single-piece graphene-modified lead-selective electrodes.

The procedure of the single-piece electrode preparation is based on incorporating graphene powder directly into the membrane matrix. The fabrication process was designed to be as simple as possible and takes less time compared to the preparation of solid-contact electrodes with separate layers. The schematic representation of the preparation procedure is presented in Figure 2. The procedure began with the preparation of GC disk electrodes’ surfaces by polishing them with alumina slurries and rinsing with water and methanol.

Membranes were cast onto a surface of GC electrodes using a drop-casting technique, with volumes of 20, 40 and 60 µL. Three electrodes, representing each group, were prepared: GC/GR(20)/Pb^2+^ISM, GC/GR(40)/Pb^2+^ISM and GC/GR(60)/Pb^2+^ISM.

The proposed electrode design, based on graphene incorporation into the membrane matrix, is novel among lead-selective potentiometric sensors. However, the most widely used construction approach involves solid-contact electrodes. Omitting the solid-contact layer preparation step makes sensor fabrication simpler and faster.

### 2.3. Methods

The main electrochemical technique used for the examination and validation of the designed sensors was potentiometry. Experiments conducted to determine the analytical parameters were performed using a 16-channel mV-meter (Lawson Labs, Inc., Malvern, PA, USA). The potentiometric response towards lead(II) ions was examined in standard Pb(NO_3_)_2_ solutions of 1.0 × 10^−8^ to 1.0 × 10^−2^ M concentration with addition of KNO_3_. Potentiometric measurements were conducted against the two-coated reference electrode—Ag/AgCl electrode with a 3 M KCl inner solution and 0.01 M CH_3_COOLi solution (6.0726.100 Metrohm, Herisau, Switzerland)—in the presence of an auxiliary electrode (platinum wire).

The chronopotentiometry measurements were carried out with the use of an Autolab General Purpose Electrochemical System (AUT302N.FRA2-AUTOLAB, Metrohm Autolab, Barendrecht, The Netherlands) with NOVA 2.1. software. Designed single-piece ISEs were tested as working electrodes in a three-electrode cell with the two-coated reference electrode—Ag/AgCl electrode with a 3 M KCl inner solution and 0.01 M CH_3_COOLi solution (6.0726.100 Metrohm, Herisau, Switzerland)—in the presence of an auxiliary electrode (glassy carbon electrode). Tests were conducted in the 1.0 × 10^−2^ M standard Pb(NO_3_)_2_ solution. A constant current of +1 nA was applied to the working electrode for 60 s, followed by a −1 nA current for another 60 s according to the procedure proposed by Bobacka [31].

The wettability measurements were carried out using a Theta Lite microscope by Biolin Scientific (Gothenburg, Sweden). A water droplet of 5 µL was discharged onto the membranes’ surface. The contact angle was measured using One Attension 4.0 software.

The surface morphology, microstructural distribution, and structural integrity of the intact and polymeric PVC membranes were evaluated using a Scanning Electron Microscope-Apreo 2 (Thermo Scientific, Waltham, MA, USA). To prevent surface charging effects and ensure adequate electrical conductivity under the electron beam, prepared electrode surfaces were sputter-coated with a thin, uniform layer of gold under vacuum. Micrographs of the PVC membrane matrices were recorded inside the electron microscope sample chamber at varying magnifications ranging from 5000× to 10,000× under an optimized accelerating voltage.

## 3. Results

### 3.1. Electrical Properties

The electrical properties of the designed membranes were tested using chronopotentiometry. This technique was applied to determine electrical capacitance and resistance parameters. The electrical parameters of the graphene-based membranes were compared with the properties determined for the lead-selective membranes without any additives.

**Figure 3:**
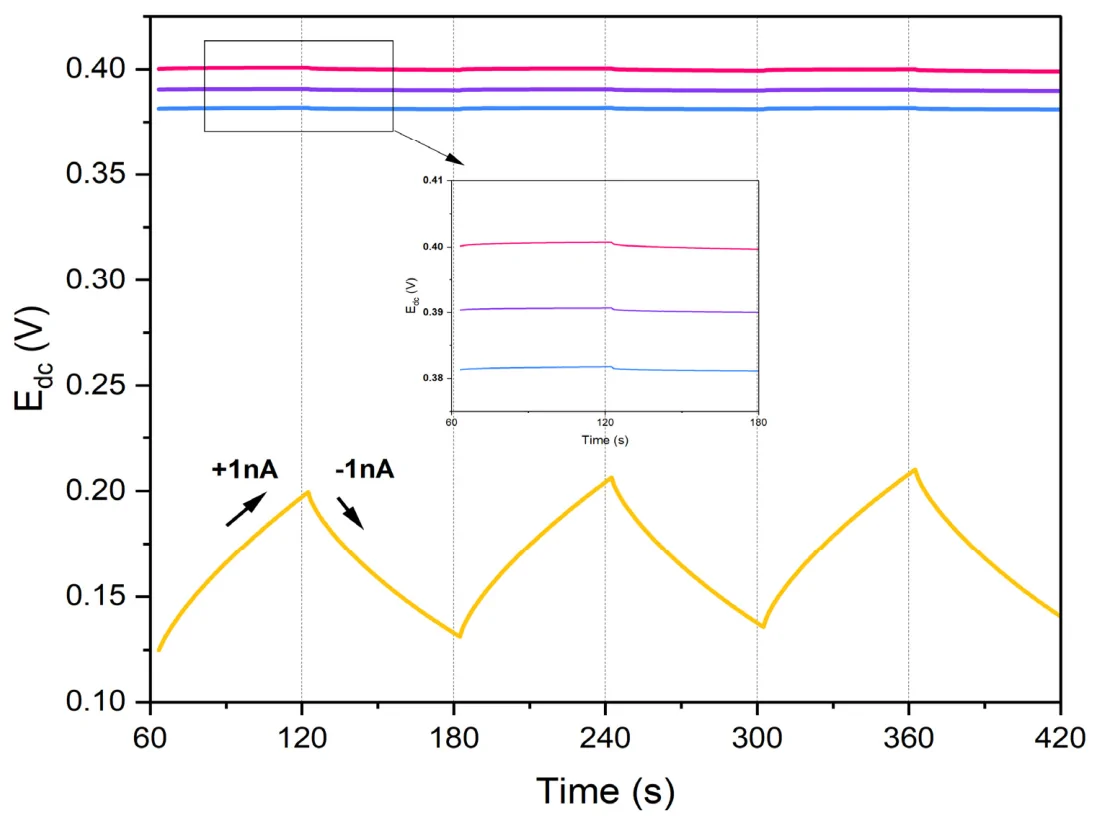
Chronopotentiograms recorded for the unmodified Pb(II)-selective electrode, GC/Pb^2+^ISM (yellow), and graphene-modified electrodes prepared with sensing cocktail volumes of 20 µL (pink), 40 µL (purple), and 60 µL (blue). Measurements were carried out in a 1.0 × 10^−2^ M Pb(NO_3_)_2_ solution using an alternating current of ±1 nA.

In chronopotentiometry technique, a current of positive and negative signs, alternately, was passed through the three-electrode cell with 1.0 × 10^−2^ M lead(II) nitrate as an electrolyte. The value of the current was set at 1 nA. The achieved potential–time graph is presented in Figure 3.

According to obtained chronopotentiograms, the potential drift of modified electrodes given by the dE/dt ratio equaled to 6.3 × 10^−6^ V/s, 4.5× 10^−6^ V/s, and 6.6 × 10^−6^ V/s for the GC/GR(20)/Pb^2+^ISM, GC/GR(40)/Pb^2+^ISM and GC/GR(60)/Pb^2+^ISM electrodes respectively. Using the equation [29]

(1) $$C=I\cdot \frac{dt}{dE_{dc}}$$

where *I* stands for current, and $\frac{dt}{dE_{dc}}\;$stands for potential drift, the electrical capacitance was calculated for the linear part of the slope.

The resistance parameter was calculated with the use of the equation

(2) $$R=\frac{I}{dE_{dc}}$$

where *dE_dc_* stands for the potential jump between two steps, and *I* is the current applied during the experiment. The highest value of electric capacitance was obtained for electrodes GC/GR(60)/Pb^2+^ISM and GC/GR(40)/Pb^2+^ISM. Electrode GC/GR(40)/Pb^2+^ISM was selected for further validation due to its good electrical properties and minimalization of chemical use in the electrode’s preparation process. The obtained results were compared with an electrode with an unmodified membrane formed from 40 µL of sensing cocktail. The exact values of capacitance and resistance are presented in Table 1.

Using Equation (1), the electrical capacitance was calculated from the slope of the chronopotentiometric response. The obtained capacitance values reached 252 µF, 324 µF, and 327 µF for GC/GR(20)/Pb^2+^ISM, GC/GR(40)/Pb^2+^ISM, and GC/GR(60)/Pb^2+^ISM, respectively. In contrast, the unmodified electrode exhibited a capacitance of only 0.86 µF. The substantial increase in capacitance after graphene incorporation demonstrates the beneficial effect of graphene nanostructures on charge storage and ion-to-electron transduction processes within the ion-selective membrane.

The resistance values, calculated from Equation (2), were 0.15 MΩ, 0.12 MΩ, and 0.13 MΩ for GC/GR(20)/Pb^2+^ISM, GC/GR(40)/Pb^2+^ISM, and GC/GR(60)/Pb^2+^ISM, respectively, whereas the unmodified membrane exhibited a significantly higher resistance of 1.90 MΩ. These results indicate that the incorporation of graphene markedly improves charge-transfer properties and facilitates electron transport across the membrane/electrode interface.

Among the investigated systems, the GC/GR(40)/Pb^2+^ISM electrode exhibited the lowest potential drift and resistance while maintaining a capacitance comparable to that of the thickest membrane. Therefore, the membrane prepared from 40 µL of sensing cocktail was selected for further studies. This choice provided favorable electrical characteristics while reducing material consumption and simplifying sensor fabrication.

Graphene nanostructures incorporated directly into the membrane matrix create conductive pathways that promote charge redistribution and enhance the interfacial capacitance. Consequently, the membrane acts not only as a selective recognition phase but also as an efficient transduction medium, enabling stable ion-to-electron conversion and improved electrochemical performance.

### 3.2. Wettability

**Figure 4:**
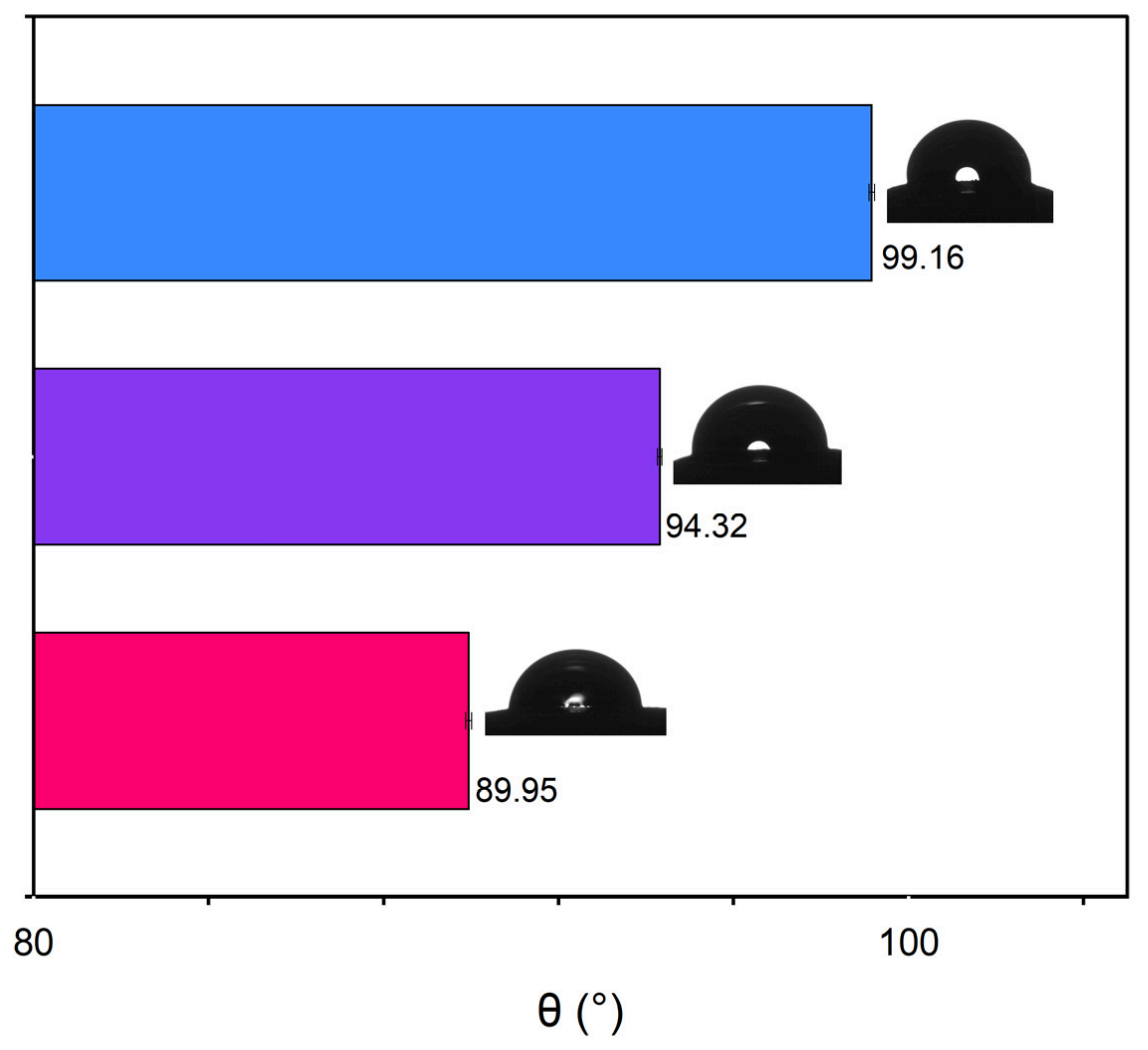
Contact angle values determined for Pb(II)-selective membranes prepared from different sensing cocktail volumes: 20 µL (pink), 40 µL (purple), and 60 µL (blue).

The wettability test was performed with the use of a tensiometer, which allows for the measurement of the contact angle of materials. A water droplet of 5 µL was discharged from a syringe onto the electrode covered with the lead-selective membrane. The results are presented in Figure 4.

The measurements were carried out to evaluate the influence of the volume of sensing cocktail used for membrane deposition (20, 40, and 60 µL) on the surface properties of the sensing layer and to identify the most suitable membrane volume for further sensor evaluation. The membranes prepared from 20, 40, and 60 µL of membrane cocktail exhibited contact angles of 89.95°, 94.32°, and 99.16°, respectively.

The results indicate that increasing the volume of sensing cocktail used for membrane deposition, and consequently the amount of membrane material deposited on the electrode, resulted in a gradual increase in hydrophobicity, as evidenced by the larger contact angle values.

From the perspective of potentiometric sensing, high contact angle values are advantageous because they reflect a more hydrophobic membrane surface, which suppresses water uptake and minimizes the formation of an undesirable aqueous layer at the membrane/electrode interface. Such behavior contributes to improved potential stability and enhanced long-term performance of all-solid-state ion-selective electrodes. Among the investigated membranes, the 60 µL membrane exhibited the highest contact angle (99.16°), indicating the most pronounced hydrophobic character and suggesting more favorable conditions for stable ion-to-electron transduction.

Wettability testing confirmed the selection of the GC/GR(40)/Pb^2+^ISM sensor for further validation, owing to its superior hydrophobic character. Although the 60 µL membrane exhibited the highest contact angle, the 40 µL configuration provided a favorable balance of surface properties and electrical performance and was therefore selected for further validation.

### 3.3. Ionic Response

**Figure 5:**
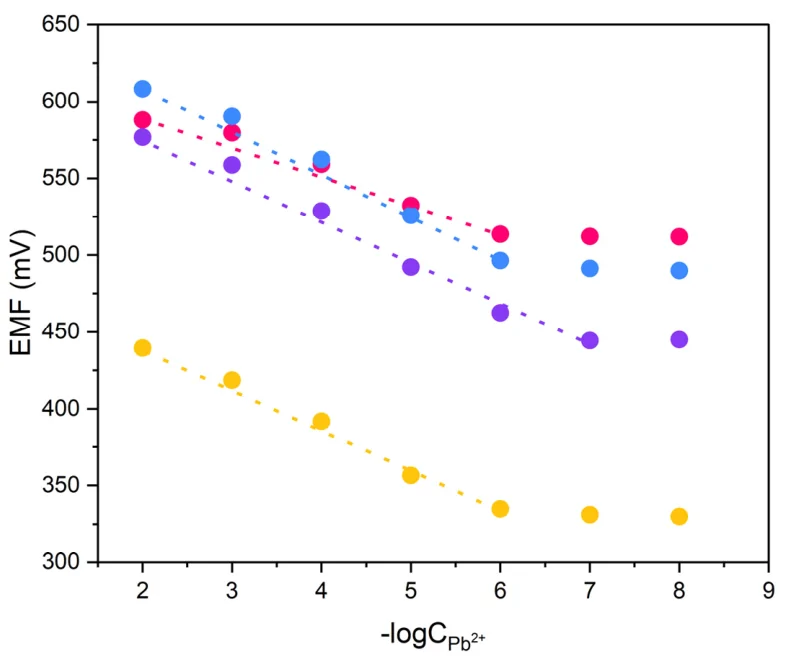
Representative potentiometric calibration curves of the unmodified Pb(II)-selective electrode, GC/Pb^2+^ISM (yellow), and graphene-modified electrodes prepared with sensing cocktail volumes of 20 µL (pink), 40 µL (purple), and 60 µL (blue).

Ionic response was examined using the potentiometric method. The sensors’ response towards lead(II) ions was evaluated based on the parameters of calibration curves. The first calibration was conducted after 24 h from the beginning of electrodes’ conditioning in 1.0 × 10^−3^ M standard Pb(NO_3_)_2_ solution. Subsequent calibrations were carried out after conditioning the electrodes in 1.0 × 10^−3^ M standard Pb(NO_3_)_2_ solution for 1 h. The EMF measurements were performed in solutions with increasing lead(II) ion concentration with addition of 1.0 × 10^−2^ M of KNO_3_ to maintain ionic strength. The exemplary potentiometric response (the relationship between EMF and -logC_Pb2+_ value) of the designed sensors is presented in Figure 5.

A linear dependence between the EMF and the negative logarithm of Pb(II) concentration was observed over a broad concentration range, demonstrating the proportional relationship between the electrode potential and Pb(II) activity. Among the investigated membrane thicknesses, the electrode prepared with 40 µL of membrane cocktail exhibited the widest linear response range and the most favorable analytical characteristics. In contrast, thinner (20 µL) and thicker (60 µL) membranes provided narrower linear ranges, indicating that membrane thickness significantly affects the transport and distribution of ionic species within the membrane phase.

The superior performance of the 40 µL membrane suggests that this thickness provides an optimal balance between ion transport and electrical properties of the membrane matrix. The obtained results confirm that graphene incorporation does not compromise the selective recognition of Pb(II) ions and that the proposed ion-selective membrane enables stable and proportional potentiometric responses over a wide concentration range.

At very low concentrations the lack of potentiometric response can be observed. It is caused by leaching of the primary ion from the membrane into the solution. The membrane always contains a small amount of free ion or ionophore salt, which can diffuse out into the sample.

### 3.4. Reproducibility

The comparison of electrodes’ parameters within each group provides information about the reproducibility of potentiometric response. The indicating factor of reproducibility was the standard deviation value, calculated based on the results obtained for each group (n = 3 items of electrodes within one group). The results are presented in Table 2. The measurements confirmed a good reproducibility of graphene-based electrodes in comparison to ones without additives, and that the registered potential was repetitive.

All investigated electrodes exhibited satisfactory repeatability, as demonstrated by the relatively low standard deviation values. In particular, the graphene-modified electrodes showed good reproducibility of both the calibration slope and the standard potential, indicating that the membrane preparation procedure provided consistent sensor performance.

Among the investigated membrane thicknesses, the GC/GR(40)/Pb^2+^ISM electrode exhibited the most favorable analytical characteristics. The average sensitivity amounted to 30.3 ± 0.6 mV dec^−1^, which was the closest to the theoretical Nernstian slope for divalent ions (29.6 mV dec^−1^). In addition, this electrode provided the broadest linear response range (1.0 × 10^−7^–1.0 × 10^−2^ M) and the lowest detection limit among all investigated membrane configurations.

The standard deviation values obtained for the GC/GR(40)/Pb^2+^ISM electrode were relatively low (±0.6 mV dec^−1^ for sensitivity and ±6 mV for the standard potential), confirming good repeatability of the fabrication procedure and excellent reproducibility of the potentiometric response. These results, together with the favorable electrical properties determined by chronopotentiometry, further justify the selection of the 40 µL membrane for subsequent investigations.

The results obtained from potentiometric calibration further confirmed the selection of the 40 µL membrane for subsequent studies. In addition to exhibiting the most favorable electrical properties, this membrane thickness provided a near-Nernstian response, the widest linear range, the lowest detection limit, and excellent reproducibility, as reflected by the small standard deviation values. Therefore, the GC/GR(40)/Pb^2+^ISM electrode was considered the optimal sensor configuration.

### 3.5. Potential Stability

The stability of the potentiometric response was tested during the long-time (15 h) measurement. Electrodes of each group were tested in the 1.0 × 10^−3^ M standard lead(II) ion solution during the potentiometric measurement, versus the reference electrode. The potential stability of the sensors was assessed using the potential drift parameter, which is calculated as potential difference/time ratio.

**Figure 6:**
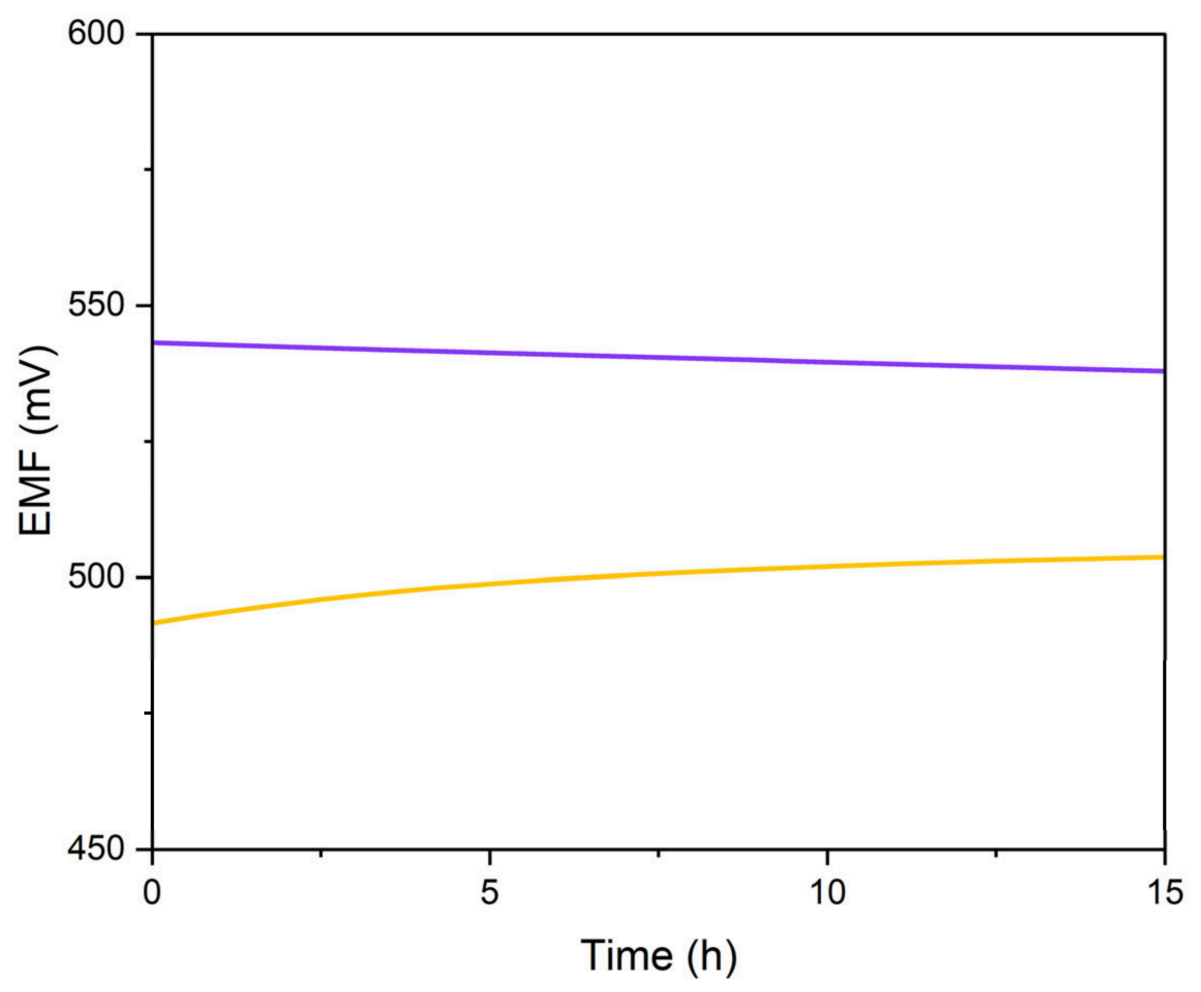
Long-term potential stability of Pb(II)-selective electrodes recorded over 15 h in 1.0 × 10^−3^ M Pb(II) solution. The potential response of the unmodified electrode, GC/Pb^2+^ISM (yellow), is compared with that of the graphene-modified electrode with the optimized membrane thickness, GC/GR(40)/Pb^2+^ISM (purple).

The long-term potential stability measurements revealed a significant influence of graphene incorporation on the stability of the Pb(II)-selective electrodes. As shown in Figure 6, the unmodified GC/Pb^2+^ISM electrode exhibited a potential drift of 0.81 mV h^−1^, whereas the graphene-containing electrode (GC/GR(40)/Pb^2+^ISM) showed a substantially lower drift of 0.35 mV h^−1^. The nearly two-fold reduction in the drift value demonstrates the beneficial effect of graphene on the stability of the potentiometric response.

The improved potential stability can be attributed to the excellent electrical properties of graphene and its ability to facilitate efficient charge transfer within the membrane matrix phase. The presence of graphene nanostructures enhances the capacitance of the sensing layer and promotes more effective ion-to-electron transduction, resulting in a more stable interfacial potential. Furthermore, the hydrophobic nature of graphene may contribute to reducing water accumulation at the membrane/electrode interface, which is one of the major factors responsible for potential instability in all-solid-state ion-selective electrodes.

These results indicate that the graphene-based ion-selective membrane provides a more stable electrochemical environment, leading to improved long-term performance of the Pb(II)-selective sensor.

### 3.6. Potential Reversibility

**Figure 7:**
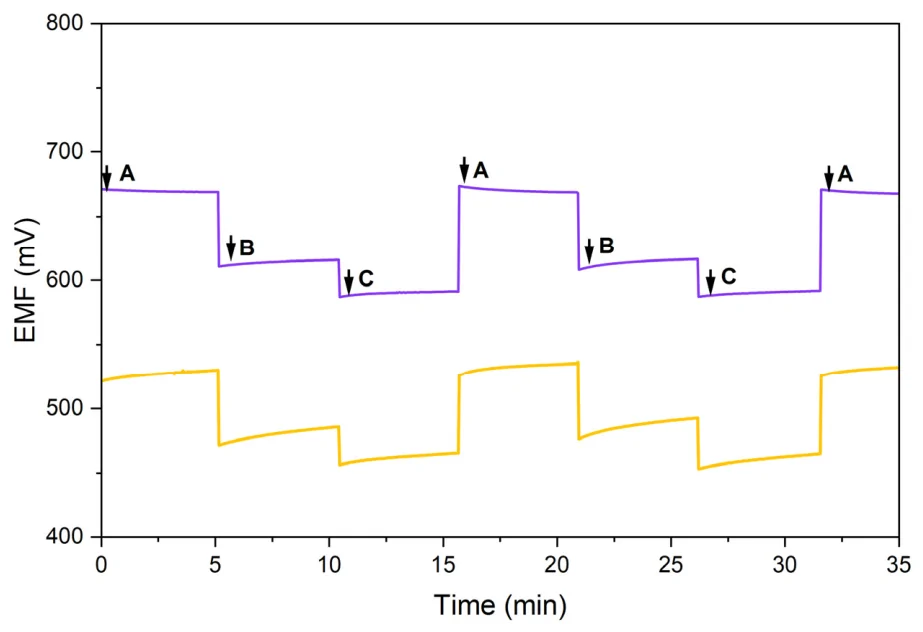
Potential reversibility of Pb(II)-selective electrodes during cyclic measurements in 1.0 × 10^−2^ M Pb(NO_3_)_2_ (A), 1.0 × 10^−4^ M Pb(NO_3_)_2_ (B), and 1.0 × 10^−5^ M Pb(NO_3_)_2_ (C) solutions. The response of the unmodified GC/Pb^2+^ISM electrode (yellow) was compared with that of the graphene-modified electrode with the optimized membrane thickness, GC/GR(40)/Pb^2+^ISM (purple).

The main goal of this experiment was to evaluate the reversibility of the potential and the stability of the potentiometric time after exchanging the analyzed solutions. For this purpose, using a 1.0 × 10^−2^ M Pb(NO_3_)_2_ (A) and a 1.0 × 10^−4^ M Pb(NO_3_)_2_ (B) and 1.0 × 10^−5^ M Pb(NO_3_)_2_ (C) solutions, the potential was measured alternately for 5 min, as presented in Figure 7.

Both electrodes exhibited reproducible and reversible responses upon successive changes in Pb(II) concentration. However, distinct differences in signal stability were observed between the unmodified and graphene-modified sensors. The GC/Pb^2+^ISM electrode (yellow) showed pronounced potential drift and a slower stabilization after each concentration change, whereas the graphene-containing electrode with the optimized membrane thickness, GC/GR(40)/Pb^2+^ISM (purple), exhibited a more stable and reproducible response with smaller potential fluctuations. Calculated potential drift values were in the range of 1.7–2.8 mV/min for non-modified sensors, compared to 0.41–1.0 mV/min for graphene-based ISM sensors.

The improved reversibility and signal reproducibility of the graphene-modified electrode indicate more efficient ion-to-electron transduction and enhanced stability of the membrane/electrode interface. Moreover, the graphene-modified electrode repeatedly returned to the characteristic potential values corresponding to each Pb(II) concentration, indicating good reversibility of the potentiometric response. These results confirm the robustness of the graphene-containing membrane architecture and its suitability for prolonged potentiometric measurements under varying analytical conditions.

The presence of graphene nanostructures facilitates charge redistribution within the membrane matrix phase and contributes to the stabilization of the interfacial potential, resulting in improved repeatability and reversibility of the potentiometric response.

### 3.7. Water-Layer Test

**Figure 8:**
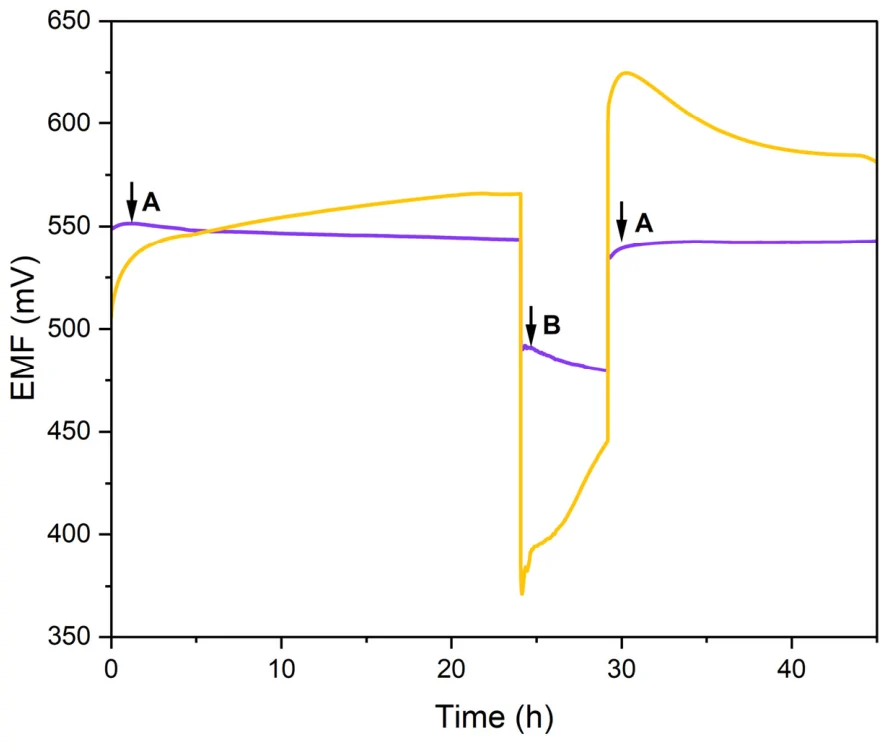
Water-layer test. The EMF response was recorded during sequential immersion in 1.0 × 10^−2^ M Pb(NO_3_)_2_ solution (A, 24 h), 1.0 × 10^−2^ M NaNO_3_ solution (B, 6 h), and again in 1.0 × 10^−2^ M Pb(NO_3_)_2_ solution (A, 15 h) for the unmodified GC/Pb^2+^ISM electrode (yellow) and GC/GR(40)/Pb^2+^ISM (purple).

The possibility of the formation of a water layer was evaluated during the water-layer test. The water-layer test was conducted according to the procedure presented by Fibbioli et al. [32]. The purpose of the experiment was to investigate whether graphene incorporated directly into the ion-selective membrane could suppress the formation of an undesirable aqueous layer. During the test, the EMF was continuously recorded while the electrodes were sequentially immersed in 1.0 × 10^−2^ M Pb(NO_3_)_2_ solution (A) for 24 h, followed by 1.0 × 10^−2^ M NaNO_3_ solution (B) for 6 h, and finally returned to solution A for an additional 15 h as presented in Figure 8.

The unmodified GC/Pb^2+^ISM electrode (yellow) exhibited a pronounced potential drift after re-immersion in the primary ion solution, corresponding to a drift value of 2.84 mV h^−1^ during the final 15 h period. Such behavior indicates the presence of an aqueous layer trapped at the membrane/electrode interface, which leads to uncontrolled ion exchange and deterioration of the potential stability. In contrast, the graphene-modified electrode with the optimized membrane thickness, GC/GR(40)/Pb^2+^ISM (purple), displayed a considerably lower potential drift of only 0.20 mV h^−1^. Moreover, the potential rapidly recovered after switching back to the Pb(NO_3_)_2_ solution, indicating effective suppression of water-layer formation.

The improved behavior of the graphene-containing membrane can be attributed to the hydrophobic nature of graphene and its favorable interaction with the membrane matrix, which limits water accumulation at the buried interface. Consequently, the graphene-assisted membrane matrix provides a more stable ion-to-electron transduction pathway and contributes to enhanced long-term stability of the Pb(II)-selective electrode.

The results of the water-layer test are in agreement with the wettability measurements. The contact angle studies demonstrated that the membrane surface exhibited pronounced hydrophobic character, with the optimized graphene-containing membrane showing a contact angle of 94.32°. The enhanced hydrophobicity of the membrane effectively limits water uptake and suppresses the accumulation of an aqueous layer at the membrane/electrode interface. As a result, the graphene-modified electrode displayed significantly lower potential drift and rapid recovery of the initial EMF after returning to the primary ion solution. These findings indicate that the direct incorporation of graphene into the polymeric membrane not only improves charge-transfer properties but also contributes to maintaining a stable interfacial environment, which is essential for long-term potentiometric performance.

### 3.8. Redox Sensitivity

**Figure 9:**
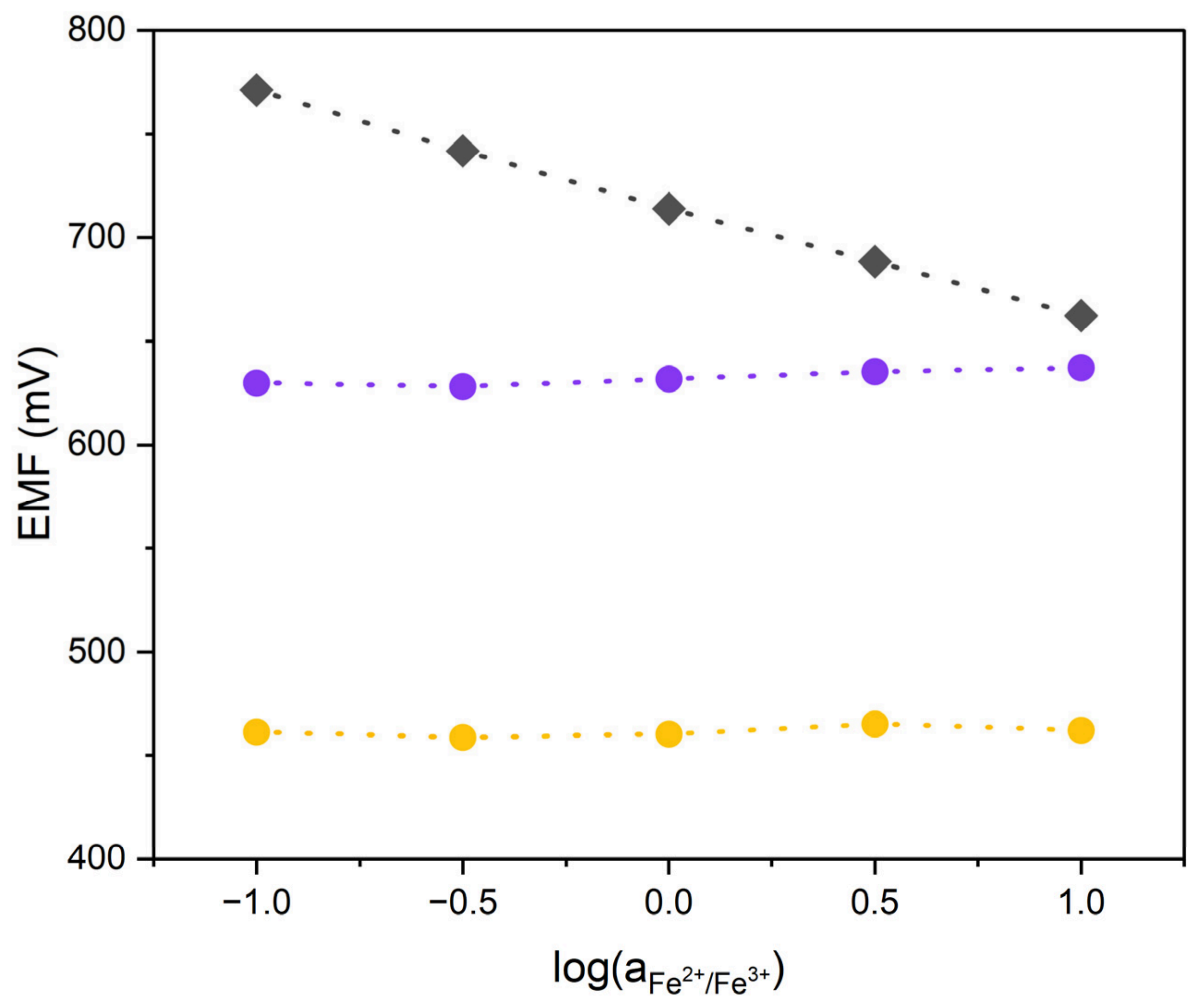
Redox sensitivity test performed using the Fe^2+^/Fe^3+^ redox couple. The response of the bare glassy carbon electrode (black) exhibited a Nernstian dependence on the activity ratio of the redox species, whereas the unmodified Pb(II)-selective electrode, GC/Pb^2+^ISM (yellow), and the graphene-modified electrode with the optimized membrane thickness, GC/GR(40)/Pb^2+^ISM (purple), maintained stable potentials independent of the redox conditions.

Graphene as carbon nanomaterial is known to be a redox-sensitive substance. The presence of redox couples in the analyzed solutions may affect the potentiometric response if the electrode is sensitive to changing redox conditions. This phenomenon is not desirable. The redox sensitivity test was conducted in order to confirm that obtained lead(II)-selective membranes with graphene are not sensitive to changing redox conditions. Potentiometric measurements were carried out in solutions with fixed concentrations of FeCl_2_ and FeCl_3_ as the redox couple, at Fe^2+^/Fe^3+^ concentration ratios corresponding to logarithmic values of −1, −0.5, 0, 0.5, and 1. Figure 9 presents the potential response of the electrode as a function of varying redox conditions.

As expected, the bare glassy carbon electrode exhibited a Nernstian response proportional to the activity ratio of the Fe^2+^/Fe^3+^ redox couple, confirming its sensitivity to changes in the redox potential of the solution. In contrast, both the unmodified Pb(II)-selective electrode and the graphene-modified electrode with the optimized membrane thickness displayed nearly constant potentials throughout the entire experiment, indicating that their responses were independent of the redox activity of the solution.

These results demonstrate that the incorporation of graphene into the polymeric membrane does not introduce undesired redox sensitivity. The ion-selective membrane effectively isolates the transduction layer from direct interactions with redox-active species present in the sample, preserving the selective potentiometric response toward Pb(II) ions. Consequently, the developed graphene-containing electrode is expected to maintain stable operation in complex environmental samples, where oxidation–reduction conditions may vary significantly.

The membrane components and the ionophore govern the sensing mechanism, while graphene acts primarily as a charge-transfer mediator without participating directly in redox processes occurring in the bulk solution. Therefore, graphene enhances the electrical properties of the membrane phase without compromising the selectivity and stability of the potentiometric response.

### 3.9. Surface Morphology

**Figure 10:**
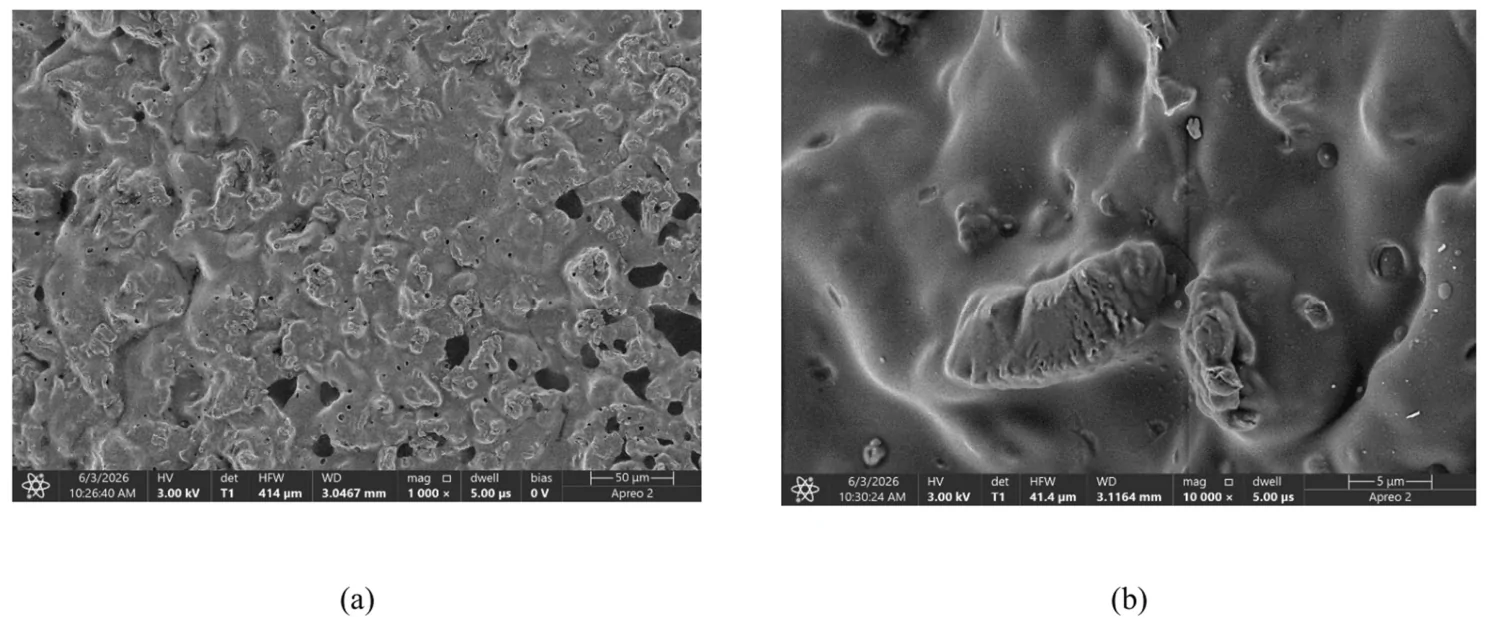
SEM scans of graphene-containing Pb^2+^-selective membrane: (**a**) 1000x (**b**) 10,000x.

Scanning electron microscopy (SEM) was employed to evaluate graphene distribution in the polymeric membrane. Representative micrographs of the optimized membranes (prepared by depositing 40 µL of ISM onto GC electrodes) are presented in Figure 10.

SEM analysis was performed to evaluate the morphology of the graphene-containing Pb(II)-selective membrane. As shown in Figure 10a, the membrane surface was continuous and free of visible cracks or structural defects. The micrographs revealed a heterogeneous but compact surface morphology with irregular microstructural features distributed throughout the polymer matrix. No large agglomerated structures were observed in the examined areas, indicating that the membrane did not contain pronounced microscale agglomerates. The higher-magnification image (Figure 10b) confirmed the presence of embedded microstructures while maintaining the overall integrity of the membrane layer. The observed compact and continuous morphology is beneficial for achieving stable potentiometric performance and efficient charge-transfer processes.

## 4. Discussion

To assess the analytical performance of the developed graphene-based single-piece Pb(II)-selective electrode, its characteristics were compared with those of previously reported lead-selective sensors employing various transducer materials and architectures (Table 3).

The developed GC/GR(40)/Pb^2+^ISM electrode exhibited a near-Nernstian slope of 30.3 mV dec^−1^, which is in good agreement with the theoretical value for divalent ions (29.6 mV dec^−1^). Similar sensitivities have been reported for sensors modified with ionic liquids (29.8 mV dec^−1^) [31], nanocomposites (31.5 mV dec^−1^) [32], graphite contacts (31.7 mV dec^−1^) [36], and polyphenylenediamine-based systems (29.8 mV dec^−1^) [39]. Therefore, the incorporation of graphene directly into the membrane matrix does not compromise the sensitivity of the electrode and provides a response comparable to that of more complex solid-contact architectures.

The improved performance observed after graphene incorporation can be related to its role within the membrane as a conductive component facilitating ion-to-electron transduction. This interpretation is supported by the chronopotentiometric results, which showed a substantial increase in electrical capacitance and a decrease in resistance for graphene-containing membranes compared with the unmodified membrane. The optimized GC/GR(40)/Pb^2+^ISM electrode exhibited a capacitance of 324 µF and a resistance of 0.12 MΩ, whereas the corresponding unmodified GC/Pb^2+^ISM electrode showed a capacitance of only 0.86 µF and a resistance of 1.90 MΩ. The presence of graphene therefore substantially changes the electrical properties of the membrane/electrode system, providing more favorable conditions for charge transport and ion-to-electron transduction. Importantly, this effect was achieved by incorporating graphene directly into the ion-selective membrane, without introducing a separate solid-contact layer.

The linear response range of the proposed sensor (1.0 × 10^−7^–1.0 × 10^−2^ M) is comparable to that achieved by graphite-based solid-contact electrodes [36] and nanocomposite-modified electrodes [32], and superior to those reported for several conventional coated-wire and conducting polymer-based systems [34,35,38,40]. Although some electrodes reported lower concentration limits extending down to 10^−8^ or even 10^−9^ M [31,32,33], these sensors required additional functional layers or more complex architectures. Therefore, the obtained linear range confirms that the simplified single-piece design is capable of maintaining satisfactory analytical performance.

The detection limit of the graphene-based single-piece electrode (6.3 × 10^−8^ M) is among the lowest values reported for Pb(II)-selective electrodes and is comparable to that obtained for graphite-based solid-contact sensors (7.5 × 10^−8^ M) [36]. Although several sensors modified with ionic liquids, nanocomposites, or BP-C_60_ transducers achieved lower detection limits in the 10^−9^ M range [31,32,33], most previously reported systems exhibited detection limits between 10^−7^ and 10^−6^ M [34,37,38,39,40]. Therefore, the developed electrode provides high sensitivity toward Pb(II) ions despite the absence of a separate transducer layer.

Potential stability is one of the most critical parameters for all-solid-state ion-selective electrodes. The developed sensor exhibited a potential drift of 0.35 mV h^−1^, which is substantially lower than that reported for graphite solid-contact electrodes (3.2 mV h^−1^) [36] and better than the polyphenylenediamine-based system, which exhibited a drift of 1 mV per 5 min [39]. Direct comparison with some literature reports should be made cautiously because the drift values were expressed using different units, making quantitative assessment difficult. Nevertheless, the obtained value indicates good long-term stability and is consistent with the chronopotentiometric and water-layer tests, which demonstrated improved electrical properties and a substantially lower potential drift after changes in the contacting solution.

The beneficial effect of graphene was also evident from the water-layer experiment. After replacing the primary ion solution with NaNO_3_ and subsequently returning to the Pb(NO_3_)_2_ solution, the graphene-modified electrode exhibited a much lower potential drift than the unmodified electrode. This behavior indicates that the incorporation of graphene can contribute to a more stable membrane/electrode interface and reduce the effects associated with water accumulation at this interface. This observation is consistent with the wettability measurements, in which the membrane surface properties were evaluated through contact angle measurements. Thus, the improved long-term stability cannot be attributed solely to the electrical properties of graphene, but appears to result from the combined effect of graphene-assisted charge transport and stabilization of the membrane/electrode interface.

It should also be emphasized that many of the previously reported Pb(II)-selective electrodes relied on additional intermediate layers based on conducting polymers, ionic liquids, nanocomposites, fullerene derivatives, or graphite contacts [31,32,33,34,35,36]. In contrast, in the present work graphene was incorporated directly into the ion-selective membrane, eliminating the need for a separate solid-contact layer. This simplified architecture reduces the number of fabrication steps and minimizes material consumption while preserving favorable analytical and electrical characteristics. The comparison demonstrates that the proposed approach provides competitive analytical parameters while considerably simplifying the sensor design.

## 5. Conclusions

The developed graphene-modified single-piece Pb(II)-selective electrode combines a simple fabrication procedure with stable and reproducible potentiometric performance. Direct incorporation of graphene into the ion-selective membrane improved the electrical properties of the sensing phase and contributed to enhanced potential stability and resistance to water-layer formation, while preserving a near-Nernstian response toward Pb(II).

The evaluation of membrane thickness demonstrated that the volume of sensing cocktail strongly influences electrode performance. Among the tested configurations, the electrode prepared using 40 µL of membrane cocktail provided the most favorable combination of analytical and electrical properties and was therefore selected for further characterization. The optimized electrode exhibited a broad linear response range, a low detection limit, and low potential drift.

The proposed graphene-containing membrane enables ion recognition and ion-to-electron transduction within a single sensing phase, eliminating the need for a separate solid-contact layer. This simplified architecture, together with the favorable analytical performance, demonstrates the potential of graphene-modified ion-selective membranes for the development of robust single-piece potentiometric sensors for Pb(II) monitoring.

## Figures and Tables

**Table 1:** Electrical parameters of the Pb(II)-selective electrodes determined from chronopotentiometric measurement.

	Capacitance [µF]	Resistance [MΩ]
GC/GR(20)/Pb^2+^ISM	252	0.15
GC/GR(40)/Pb^2+^ISM	324	0.12
GC/GR(60)/Pb^2+^ISM	327	0.13
GC/ Pb^2+^ISM	0.86	1.90

**Table 2:** Analytical parameters of the unmodified and graphene-modified Pb(II)-selective electrodes obtained from potentiometric measurements.

Electrode Symbol	Sensitivity S ± SD [mV dec^−1^]	Standard Potential E^0^ ± SD [mV]	Linear Range [M]	Limit of Detection [M]
GC/GR(20)/Pb^2+^ISM	25.6 ± 0.5	587 ± 20	1.0 × 10^−6^– 1.0 × 10^−2^	7.1 × 10^−7^
GC/GR(40)/Pb^2+^ISM	30.3 ± 0.6	630 ± 6	1.0 × 10^−7^– 1.0 × 10^−2^	6.3 × 10^−8^
GC/GR(60)/Pb^2+^ISM	27.1 ± 0.2	650 ± 5	1.0 × 10^−6^– 1.0 × 10^−2^	8,9 × 10^−7^
GC/Pb^2+^ISM	30.5 ± 0.9	563 ± 10	1.0 × 10^−6^– 1.0 × 10^−2^	7.9 × 10^−7^

**Table 3:** Comparison of the analytical performance of the developed graphene-containing Pb(II)-selective electrode with previously reported Pb(II)-selective sensors.

Electrode Architecture	Slope [mV dec^−1^]	Linear Range [M]	Limit of Detection [M]	Potential Drift	Reference
solid-contact electrode modified with IL	29.8	1.0 × 10^−8^–1.0 × 10^−1^	4.3 × 10^−9^	0.1 mV/day	[33]
solid-contact electrode modified with NC	31.5	1.0 × 10^−8^–1.0 × 10^−2^	6 × 10^−9^	1.75 µV/min	[28]
BP-C_60_-based solid-contact electrode	28.8	1.0 × 10^−9^–1.0 × 10^−3^	6 × 10^−9^	N/R	[29]
PANI–PFOA solid-contact electrode with PAMTB additive	28.5	1.0 × 10^−6^–1.0 × 10^−3^	1.9 × 10^−7^	8.6 ± 2.9 μV/s	[34]
PEDOT(PSS) solid-contact electrode	28.58	1.0 × 10^−6^–1.0 × 10^−4^	N/R	N/R	[35]
Graphite solid-contact electrode	31.7	1.0 × 10^−7^–1.0 × 10^−2^	7.5 × 10^−8^	3.2 mV/h	[36]
Coated-wire electrode	30	1.0 × 10^−6^–1.0 × 10^−1^	6 × 10^−7^	N/R	[37]
Graphite solid-contact electrode	28	1.0 × 10^−5^–1.0 × 10^−1^	1.65 × 10^−6^	N/R	[38]
Polyphenylenediamine as solid ionophore	29.8	3.16 × 10^−6^–3.16 × 10^−2^	6.31 × 10^−7^	1 mV/5 min	[39]
Graphite solid-contact electrode	27.7	1.0 × 10^−5^–1.0 × 10^−1^	2.89 × 10^−6^	N/R	[40]
Graphene-based single-piece electrode	30.3	1.0 × 10^−7^–1.0 × 10^−2^	6.3 × 10^−8^	0.35 mV/h	this work

Abbreviation: N/R—not reported.

## Data Availability

The data presented in this study are available on request from the corresponding author.

## Abbreviations

The following abbreviations are used in this manuscript:

AAS	Atomic Absorption Spectrometry
ASV	Anodic Stripping Voltammetry
BBPA	Bis(1-butylpentyl) Adipate
BP-C60	Buckminsterfullerene–Carbon 60 (fullerene derivative)
EMF	Electromotive Force
GC	Glassy Carbon
GR	Graphene
ICP-MS	Inductively Coupled Plasma Mass Spectrometry
IL	Ionic Liquid
ISE	Ion-Selective Electrode
ISM	Ion-Selective Membrane
KTpClPB	Potassium Tetrakis(p-chlorophenyl) Borate
MIP	Molecularly Imprinted Polymer
MPC	Monolayer-Protected Cluster
NC	Nanocomposite
*o*-NPOE	2-Nitrophenyl Octyl Ether
PAMTB	Propyl 2-(acrylamidomethyl)-3,4,5-trihydroxybenzoate
PANI–PFOA	Polyaniline—Perfluorooctanoic Acid
PEDOT(PSS)	Poly(3,4-ethylenedioxythiophene) doped with Poly(styrene sulfonate)
PVC	Poly(vinyl chloride)
SC	Solid-Contact
SD	Standard Deviation
SEM	Scanning Electron Microscopy
SP	Single-Piece
THF	Tetrahydrofuran
